# Enterobactin as Part of the Oxidative Stress Response Repertoire

**DOI:** 10.1371/journal.pone.0157799

**Published:** 2016-06-16

**Authors:** Daiana R. Peralta, Conrado Adler, Natalia S. Corbalán, Enrique Carlos Paz García, María Fernanda Pomares, Paula A. Vincent

**Affiliations:** Instituto Superior de Investigaciones Biológicas (INSIBIO), CONICET-UNT and Instituto de Química Biológica “Dr. Bernabé Bloj”, Facultad de Bioquímica, Química y Farmacia, UNT. Chacabuco 461, T4000ILI -San Miguel de Tucumán, Tucumán, Argentina; Niels Bohr Institute, DENMARK

## Abstract

Microorganisms produce siderophores to facilitate iron uptake and even though this trait has been extensively studied, there is growing evidence suggesting that siderophores may have other physiological roles aside from iron acquisition. In support of this notion, we previously linked the archetypal siderophore enterobactin with oxidative stress alleviation. To further characterize this association, we studied the sensitivity of *Escherichia coli* strains lacking different components of the enterobactin system to the classical oxidative stressors hydrogen peroxide and paraquat. We observed that strains impaired in enterobactin production, uptake and hydrolysis were more susceptible to the oxidative damage caused by both compounds than the wild-type strain. In addition, meanwhile iron supplementation had little impact on the sensitivity, the reducing agent ascorbic acid alleviated the oxidative stress and therefore significantly decreased the sensitivity to the stressors. This indicated that the enterobactin-mediated protection is independent of its ability to scavenge iron. Furthermore, enterobactin supplementation conferred resistance to the *entE* mutant but did not have any protective effect on the *fepG* and *fes* mutants. Thus, we inferred that only after enterobactin is hydrolysed by Fes in the cell cytoplasm and iron is released, the free hydroxyl groups are available for radical stabilization. This hypothesis was validated testing the ability of enterobactin to scavenge radicals *in vitro*. Given the strong connection between enterobactin and oxidative stress, we studied the transcription of the *entE* gene and the concomitant production of the siderophore in response to such kind of stress. Interestingly, we observed that meanwhile iron represses the expression and production of the siderophore, hydrogen peroxide and paraquat favour these events even if iron is present. Our results support the involvement of enterobactin as part of the oxidative stress response and highlight the existence of a novel regulation mechanism for enterobactin biosynthesis.

## Introduction

*Escherichia coli* produces the archetype siderophore enterobactin, which has a trilactone backbone with three catechol moieties, exposed for iron coordination [1]. This structure allows enterobactin to tightly bind iron (Ka = 10^49^ M^-1^) and in conjunction with a specific transport system facilitates iron uptake [1, 2]. Enterobactin production is widely spread among members of the *Enterobacteriaceae* family [3]. Regulation of enterobactin synthesis and transport by the ferric uptake regulator (Fur) has been thoroughly characterized [4–7]. Under iron-rich conditions, Fur binds Fe^+2^ and acts as a negative regulator of the enterobactin system which comprises the operon *entCDEBAH* devoted to the siderophore synthesis and the *fepA*, *fepB*, *fepC*, *fepD*, *fepE*, *fepG*, *fes*, and *entS* genes involved in the enterobactin uptake and export [4–7]. There are three regulatory regions that contain two divergently oriented promoters, each having a Fur box [5] (S1 Fig). Besides the enterobactin system, Fur downregulates the transcription of several genes involved in iron metabolism, acid tolerance, virulence factor production, metabolic pathways and protection against oxidative stress [8]. When iron is limiting, Fur remains inactive and therefore enterobactin synthesis is no longer repressed. In this scenario, the small RNA RyhB gains relevance helping the cell to adapt to iron depletion [9]. RyhB increases free iron levels in several ways. For instance, it represses about 20 transcripts involved in the synthesis of non-essential iron-utilization and iron-storage proteins [10]. Furthermore, RyhB augments enterobactin production by channeling the metabolism towards the enterobactin biosynthetic pathway. This is achieved by activating the expression of *shiA* (shikimate permease) and therefore increasing the uptake of the enterobactin precursor shikimate [11]. In addition, by enhancing the translation of the *cirA* mRNA, RyhB facilitates the uptake of the enterobactin breakdown products 2,3 dihydroxybenzoic acid (DHBA) and 2,3 dihydroxybenzoic serine (DHBS), which are building blocks readily available for enterobactin synthesis [12]. Another metabolic shift that favours enterobactin synthesis driven by RyhB is the translation repression of the mRNA coding for the serine acetyl transferase CysE [13]. The blockage of cysteine synthesis from serine increases the availability of serine for enterobactin synthesis. Finally, it has been reported that RyhB directly stimulates the expression of *entCEBAH* through an unknown mechanism [13]. Since *ryhB* gene is repressed by Fur [14], the role of this sRNA in modulating the cell physiology is mostly relevant when iron is not abundant.

It has been observed in other microorganisms that siderophore synthesis can also be regulated by oxidative stress besides iron availability [15–17]. Interestingly, we previously reported that enterobactin confers protection to *E*. *coli* against the oxidative stress generated by the Pseudomonad siderophore pyochelin [18]. Furthermore, we observed that enterobactin is crucial for colony development in culture conditions that increased the oxidative stress [19]. In this work, we aimed at studying the effect of the classical stressors hydrogen peroxide (H_2_O_2_) and paraquat on *E*. *coli* growth and to further characterize the mechanism by which enterobactin reduces the oxidative stress. In addition, we investigated the potential regulation of the siderophore synthesis mediated by oxidative stress. We found that enterobactin reduces the sensitivity to both hydrogen peroxide and paraquat. In order to do that, enterobactin has to enter the cell, reach the cell cytoplasm and be linearized by the hydrolytic enzyme Fes. After iron release, the linear enterobactin molecule has three catechol moieties with reduced affinity for iron [20] and with the potential to stabilize reactive oxygen species (ROS). Corroborating the link between the siderophore and the oxidative stress response, we observed that exposure of *E*. *coli* cells to either hydrogen peroxide or paraquat increased enterobactin transcription and production even when iron was in excess.

## Results and Discussion

### Intracellular protection from H_2_O_2_ and Paraquat toxicity by enterobactin

**Fig 1:**
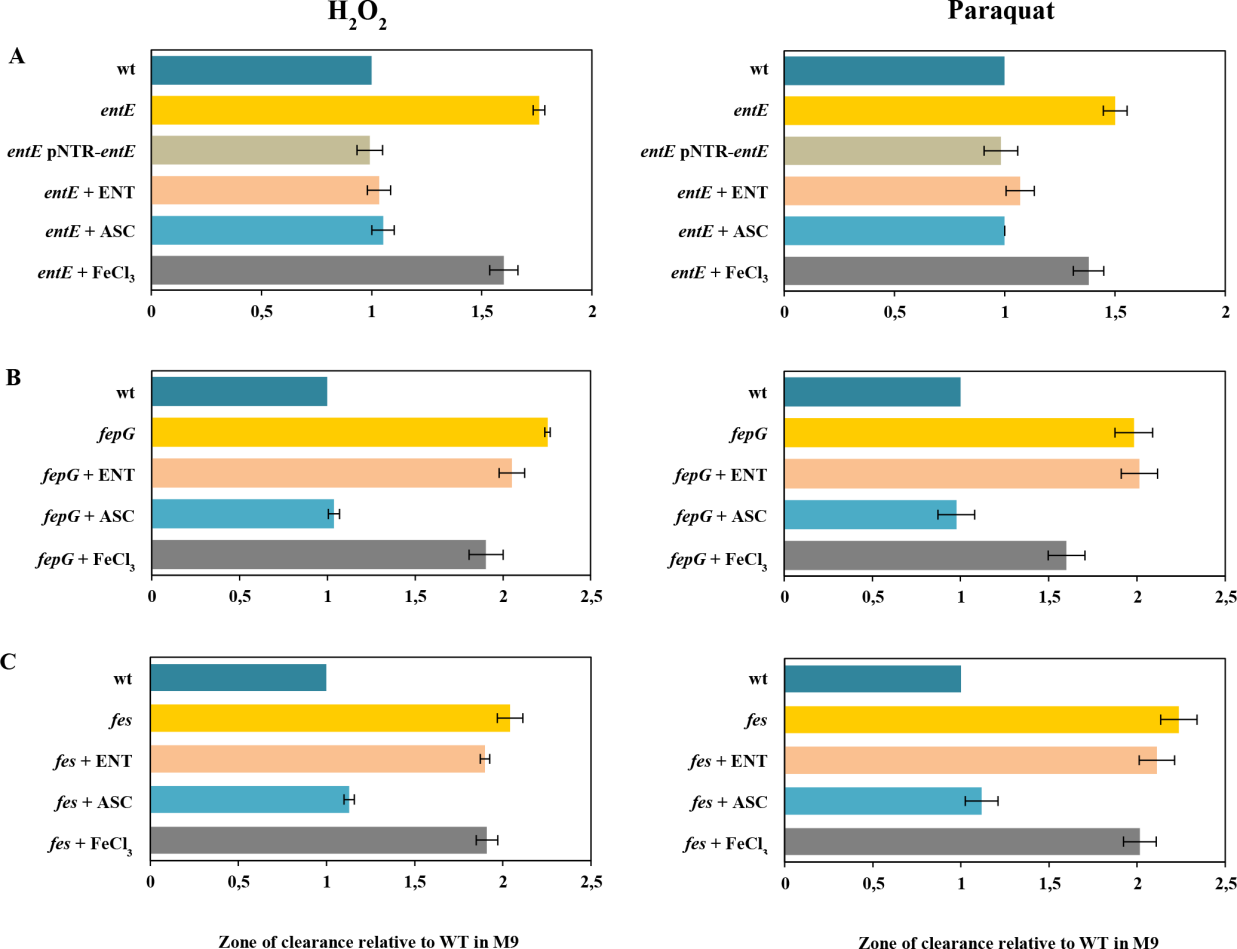
Sensitivity to hydrogen peroxide (H_2_O_2_) and paraquat and the effect of selected additives. Data is plotted as the mean values ± standard deviation (SD) of the zone of clearance obtained with strains *entE* and *entE* pNRT-*entE* (Panel A), *fepG* (Panel B) or *fes* (Panel C) relative to the zone of clearance of the wild-type strain in M9 medium. ENT, ASC and FeCl_3_ indicate medium supplementation with 1 μM enterobactin, 5 mM ascorbic acid and 100 μM FeCl_3_, respectively. Experiments were done in triplicates.

Previously, we demonstrated that enterobactin plays an important role in the oxidative stress response particularly required in the context of colony development [19]. In that work we used a set of mutants impaired in enterobactin uptake, hydrolysis and export to study the siderophore involvement in colony formation. We were able to conclude that enterobactin has to reach the cell cytoplasm and be hydrolyzed in order to reduce oxidative stress and allow colony formation. Furthermore, we also observed that enterobactin protected against the ROS-mediated toxic effects of the Pseudomonad siderophore pyochelin [18]. In this work, to strengthen the concept of enterobactin as part of the oxidative stress response repertoire, we characterized the sensitivity of a set of mutants to classical stressors such as hydrogen peroxide and paraquat. In Fig 1, we show the significantly higher sensitivity of a mutant impaired in enterobactin synthesis (*entE*) to both oxidants when compared with the isogenic wild-type. Furthermore, the sensitivity to hydrogen peroxide and paraquat was reverted to the same level of the wild-type by complementing the strain with a plasmid carrying the *entE* gene (pNTR-*entE*) or by supplementing the medium with either 1 μM of enterobactin or 5 mM of the reducing agent ascorbic acid (Fig 1A). S2 Fig shows representative images of *entE* and wild-type strains sensitivity to hydrogen peroxide and paraquat. Likewise, *entA*, *entB* and *entF* mutant strains showed similar sensitivity results when compared with the wild-type (S3 Fig).

In concordance with our previous results [19], we observed that *fepG* (involved in uptake) and *fes* (involved in cytoplasmic hydrolysis) mutant strains were also considerably more susceptible to H_2_O_2_ and paraquat damage compared to the wild-type (Fig 1B and 1C). Unlike with *entE* strain, addition of enterobactin to the medium did not decrease the sensitivity of the *fepG* and *fes* strains. This indicates that the enterobactin-mediated protection against oxidative stress involves the same mechanism observed with enterobactin in allowing colony formation. In both cases, enterobactin is only able to alleviate the oxidative stress after being internalized and hydrolyzed in the cytoplasm. On the contrary, ascorbic acid supplementation non-specifically reduced the sensitivity of *fepG* and *fes* mutant strains to the same level of the wild-type. As expected, additional mutants impaired in enterobactin uptake (*i*.*e*. *fepB*, *fepC* and *fepD)* displayed the same sensitivity phenotype as the *fepG* strain when compared with the wild-type (S4 Fig). Since ROS protection depend in part on some detoxifying enzymes that require iron [21], we considered necessary to rule out iron deficiency as a cause for the differential sensitivity between the mutants in the enterobactin system and the wild-type. For that, we supplemented the media with 100 μM FeCl_3_ and observed only a slightly reduction in sensitivity to H_2_O_2_ and paraquat with all mutants tested (Fig 1, S2 and S3 Figs). Given that mutants remained more sensitive than the wild-type even with iron addition we conclude that iron availability is not accountable for the increased sensitivity phenotype. This is consistent with our previous work [19], where we demonstrated that iron availability is not related to the impaired colony development of *entE* cells. It is also worth noting that *E*. *coli* possesses alternative iron uptake systems that provide iron to cells even in the absence of enterobactin [19].

Taking together, these results support the concept that enterobactin plays a role in the defense against various sources of oxidative stress independently of its ability to facilitate iron uptake. Similar observations have been made in *Salmonella* Typhimurium [22], where it was demonstrated that salmochelin and enterobactin once inside bacterial cells, protect against ROS damage in a macrophage invasion model. In addition, this work suggests that catechol-dependent protection against oxidative stress is not linked to iron availability. Interestingly, it was recently reported a new level of enterobactin involvement in protection against oxidative stress [23]. The iron-free form of the siderophore was shown to inhibit the host neutrophilic antibacterial enzyme myeloperoxidase, therefore avoiding the production of the potent oxidizing agent hypohalous acid. Furthermore, authors showed that a *fepA* mutant, despite being unable to uptake the iron-enterobactin complex, has an increased ability to colonize the inflamed gut due to the overproduction of enterobactin [23]. There are numerous reports that associate siderophores with oxidative stress protection. However, many of those reports explain this role of siderophores based on their ability to chelate iron and therefore prevent Fenton reaction. For example, in *Azotobacter vinelandii*, the siderophores protochelin and azotochelin were shown to protect against paraquat [15]. Similarly, in *Aspergillus fumigatus* it was observed that the intracellular siderophore ferricrocin protects against oxidative stress through an equivalent mechanism [24]. Even though siderophore-mediated protection against oxidative stress may involve sequestering iron or other varied mechanisms, the amount of data gathered until now constitute compelling evidence of their participation in oxidative stress response.

### Oxidative stress induces Enterobactin production in *E*. *coli*

**Fig 2:**
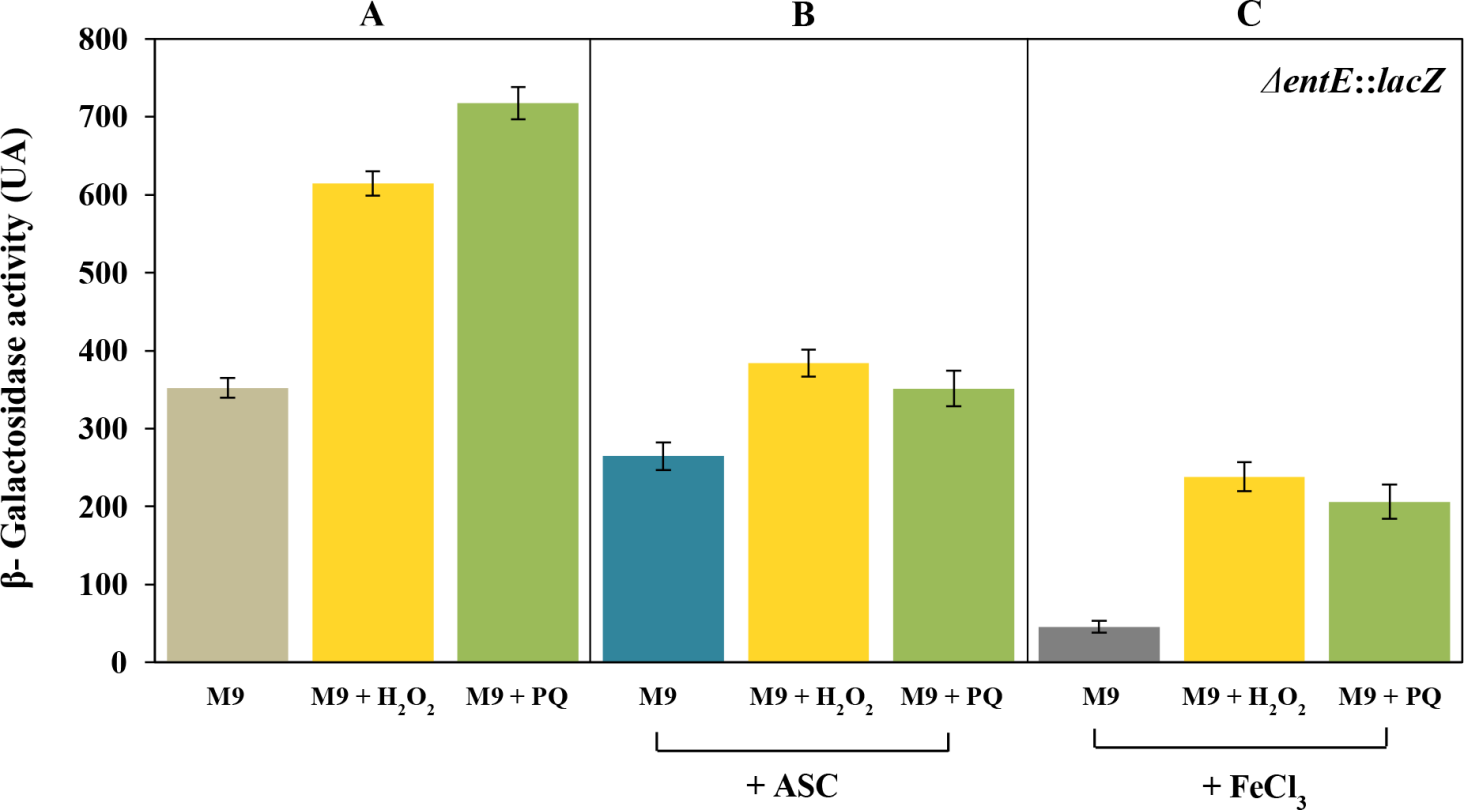
Effect of oxidative stress on *entE* expression. Panel A shows the effect of 1mM hydrogen peroxide (H_2_O_2_) or 100 μM paraquat (PQ) on *entE* expression. Panel B and C display the effect of the same stressors in the presence of 5 mM ascorbic acid (ASC) and 100 μM FeCl_3_, respectively. For A, B and C, a control without addition of H_2_O_2_ or PQ was included. *entE* expression was determined by means of a β-galactosidase assay as described in the Material and Methods section. Values are means ±SD for three independent experiments.

**Fig 3:**
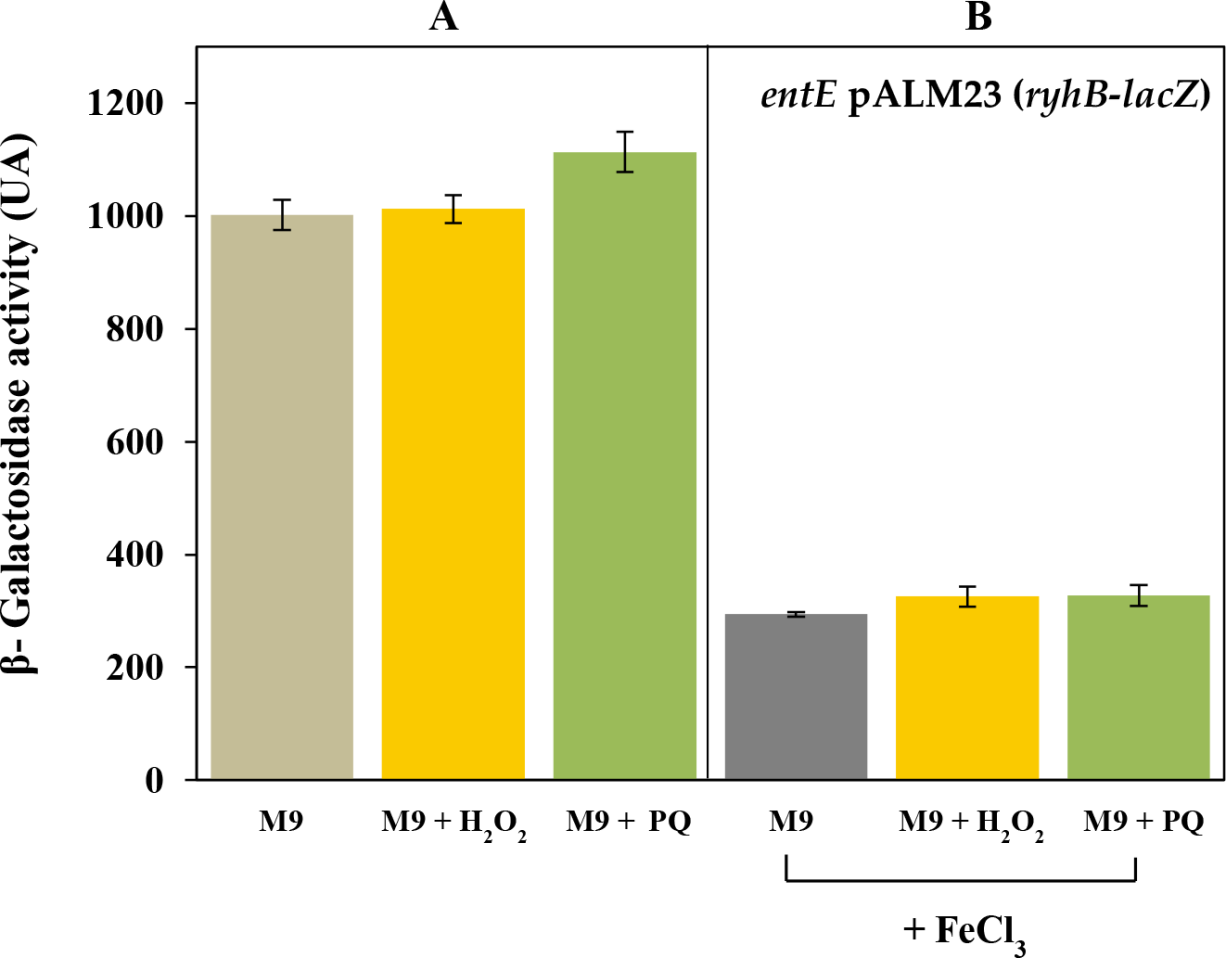
Effect of H_2_O_2_ and paraquat on iron availability. The activity of the Fur-regulated promoter *ryhB* was used as an indicator of intracellular iron levels. The expression of *ryhB* was estimated by β-galactosidase activity in an *entE* mutant transformed with the plasmid pALM23 that carries the *ryhB*-*lacZ* fusion. Panel A shows that addition of 1 mM H_2_O_2_ did not affect the activity of the promoter meanwhile 100 μM paraquat addition only slightly increased it. Panel B shows that the promoter activity is equally affected by iron addition (100 μM FeCl_3_) even in the presence of 1 mM H_2_O_2_ or 100 μM paraquat. Values are means ±SD for three independent experiments.

**Fig 4:**
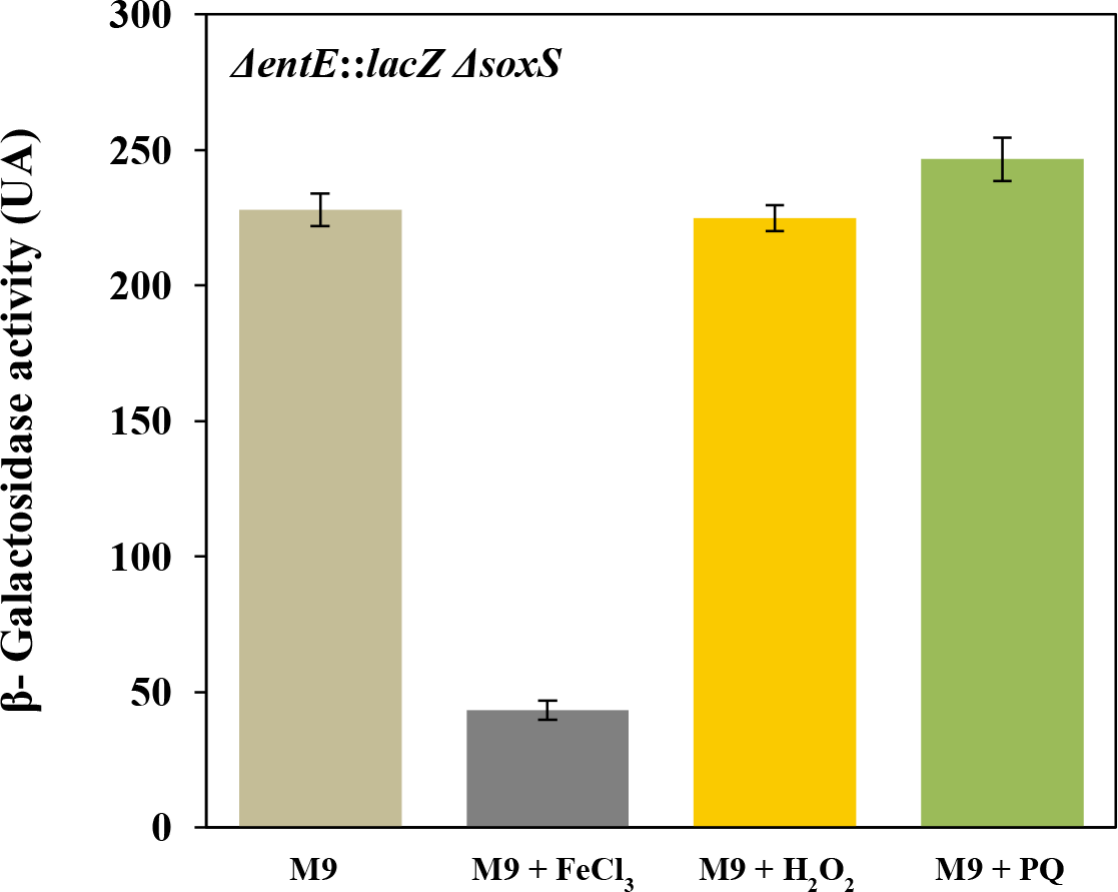
Absence of *entE* expression induction by oxidative stress in a *soxS* mutant. The expression of *entE* was estimated by β-galactosidase activity in a *ΔentE*::*lacZ* Δ*soxS* mutant as described in the Material and Methods section. In the absence of the global transcriptional regulator SoxS, H_2_O_2_ and paraquat do not induce *entE* expression. The repressive effect of iron (100 μM FeCl_3_) is preserved. Values are means ±SD for three independent experiments.

In *E*. *coli*, genes encoding enterobactin biosynthesis and transport are regulated, at a transcriptional level, by the global ferric uptake regulator (Fur) that senses intracellular iron [4–7]. In general, siderophore biosynthesis is primarily regulated by iron availability but there is evidence that their production can be influenced by other stimuli, particularly agents that mediate oxidative damage [15–17]. Since we observed that in *E*. *coli*, enterobactin plays a role in reducing reactive oxygen species [19], we decided to investigate a possible effect of oxidative stress, generated by chemical stressors, on enterobactin production. To study the regulation of enterobactin, we used a strain with a chromosomal Δ*entE*::*lacZ* transcriptional fusion grown in M9 and exposed to different oxidative conditions as shown in Fig 2. We observed that addition of 1 mM H_2_O_2_ or 100 μM paraquat in the medium, significantly increased *entE* transcription compared with the control (Fig 2A). Furthermore, the reducing agent ascorbic acid decreased the *entE* expression either in the presence or absence of added oxidants (Fig 2B). As expected, the expression of enterobactin was highly repressed by 100 μM of FeCl_3_. This constitutes additional evidence that the *entE* strain despite not producing enterobactin is able to internalize iron. However, suppression of *entE* expression by iron was not fully accomplished in the presence of H_2_O_2_ or paraquat (Fig 2C). To rule out an iron uptake imbalance in the presence of the oxidants, we estimated intracellular iron content using the Fur regulated promoter *ryhB* fused to *lacZ* as described previously [25]. Interestingly, unlike the *entE* expression, the regulation of *ryhB* by iron is not affected by the addition of oxidants (Fig 3A). Furthermore, repression of *ryhB* expression by iron was identical despite of the addition of hydrogen peroxide or paraquat (Fig 3B). Given that iron availability would not be affected in the presence of hydrogen peroxide and paraquat, our observations indicate that the oxidative stress promotes the expression of enterobactin and counteracts the repressive effect of iron (Fig 2C). Another example of siderophore biosynthesis with dual regulation (iron and oxidative stress) was described in *A*. *vinelandii*. In this bacterium catecholate siderophore production is induced by oxidative stress through the global regulator SoxS [17]. Having this in mind we evaluated the effect of oxidative stress on the expression of *entE* in a double mutant *entE soxS*. Remarkably, this strain did not increase *entE* expression upon addition of hydrogen peroxide or paraquat (Fig 4). The lack of response to oxidative stress in the double mutant *entE soxS* is suggestive of the involvement of this global regulator in enterobactin transcription. Then, the induction of *entE* expression by both paraquat and hydrogen peroxide (Fig 1A) would be consistent with the fact that the regulon SoxSR is activated not only by paraquat but also by hydrogen peroxide [26]. It has been proposed that a potential way in which cells could respond to oxidative stress and increase production of enterobactin in *Salmonella* would be to channel chorismate towards the siderophore biosynthetic pathway [22]. Authors hypothesized that AhpC, an alkyl hydroperoxidase reductase, may also act as a thiol–disulfide switch sensor that in response to oxidative stress, controls at a post-translational level, the flux of chorismate into the enterobactin biosynthetic pathway. Furthermore, another report showed that under peroxide stress, the small RNA RybA downregulates the aromatic amino acid biosynthesis which results in increased chorismate concentrations [27]. This metabolic shift favours other downstream biosynthetic pathway products like ubiquinone or enterobactin, which participate in the defense against oxidative stress in the cell [28]. However, further experiments are required to elucidate the mechanisms that mediate the up-regulation of enterobactin synthesis by oxidative stress. These mechanisms would be part of an intertwined regulatory network that allows microbes to reach a balance in iron homeostasis, oxidative stress response and other metabolic traits.

**Fig 5:**
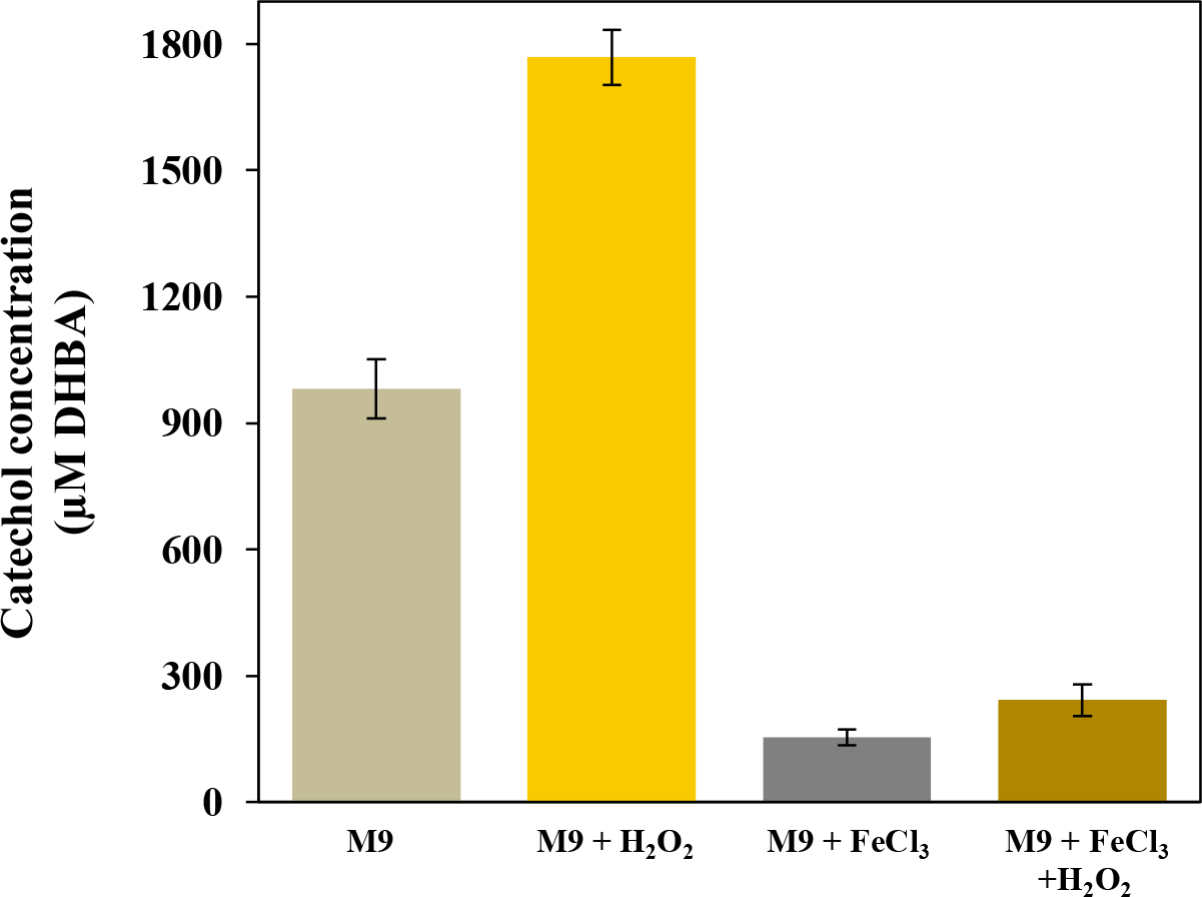
Effect of H_2_O_2_ and iron supplementation on catechol production. Quantitation of catechol levels produced by wild-type *E*. *coli* was done using Arnow assay on enterobactin-enriched extracts. Extracts were obtained from cultures grown in M9 medium supplemented with 1 mM H_2_O_2_, 25 μM FeCl_3_ or both. Results are expressed as μM equivalents by comparison to a standard curve prepared using DHBA. Values are means ±SD for three independent experiments.

To confirm that the transcriptional regulation of *entE* in the presence of iron and oxidative stress actually influences the production of the siderophore, we studied catechol levels in culture supernatants using Arnow method (see material and methods). Results demonstrate that, when cells were exposed to oxidative stress conditions (H_2_O_2_), the level of catechols produced was 80% higher than in the control (Fig 5). As expected, in the presence of iron, catechol production was significantly repressed. Interestingly, when cells were grown with supplemented iron and H_2_O_2_, catechol levels remained higher (50%) than in cultures only supplemented with iron. Previously, we showed that an *entE* mutant is unable to form colonies in agarized minimal medium [19] and that the arrested growth can be reverted by supplementation with enterobactin. In order to have a more physiological assay that reflects the level of catechols produced, we took advantage of the above-mentioned phenotype. For that, double serial dilutions of enterobactin-enriched extracts were performed and tested for their ability to restore colony formation. Table 1 shows that extracts from cultures having H_2_O_2_ were able to restore colony formation at higher dilutions when compared with extracts derived from cultures not supplemented with H_2_O_2_. In agreement with the *entE* expression and Arnow test results, we observed the same counteracting effect of iron and oxidant addition.

**Table 1:** Catechol production estimated by colony arrest reversion.

Culture conditions ^a^	Dilution ^b^
M9 medium.	1/8192
M9 + H_2_O_2_ (1 mM)	1/32768
M9 + FeCl_3_ (25 μM)	1/128
M9 + H_2_O_2_ (1 mM) + FeCl_3_ (25 μM)	1/256

^**a**^ Growth conditions of cultures from where enterobactin enriched extracts were obtained.

^**b**^ Highest dilution of extracts having enterobactin that allowed colony formation.

Similar results were observed in *Bacillus anthracis*, where catechol siderophore production increased when cells were exposed to oxidative conditions [16]. Furthermore, a recent proteomic analysis in *Acinetobacter oleivorans* showed that H_2_O_2_ treatment increased 1.9-fold the enterobactin synthetase component F, suggesting that siderophores potentially alleviate oxidative stress [29]. In this and previous studies [18, 19] we showed a strong link between enterobactin production and oxidative stress protection. A plausible mechanism that would justify our observations is that enterobactin provides the iron necessary for enzymes involved in detoxifying oxidative stress [21]. Nonetheless, *E*. *coli* has several systems that assure iron uptake and in fact a mutant impaired in enterobactin synthesis is still able to seize and use the metal. Moreover, medium supplementation with an excess of iron does not alter the oxidative stress-related phenotypes associated with enterobactin absence. Based on these observations we suggest that besides iron scavenging, enterobactin has other physiological traits that explain its role as an oxidative stress-protecting agent.

### Radical scavenging ability of Enterobactin

**Fig 6:**
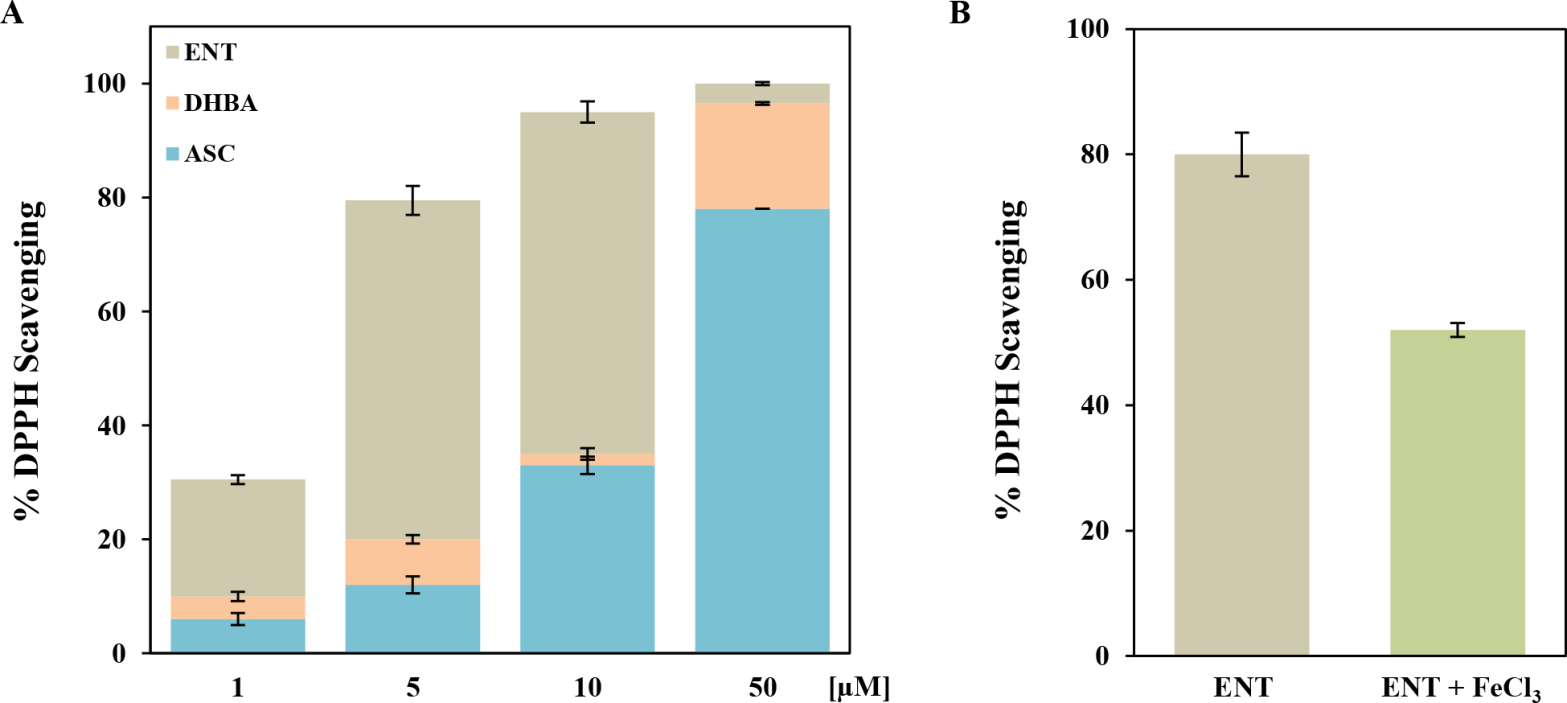
*In vitro* radical scavenging activity of enterobactin. Determination of radical scavenging ability was done following the reduction of DPPH absorbance at 517 nm (see material and methods). A) Radical scavenging ability of enterobactin (grey bars), DHBA (orange bars) and ascorbic acid (light blue bars) at different concentrations (1, 5, 10 and 50 μM). B) Effect of iron (5 μM FeCl_3_) supplementation on enterobactin (5 μM) radical scavenging activity. Values are means ±SD for three independent experiments.

Several studies have demonstrated that bacterial siderophores can act as ROS neutralizing agents [22, 30, 31]. In previous works we showed that enterobactin is able to reduce cell ROS levels generated by pyochelin or culture conditions [18]. We hypothesize two different scenarios for enterobactin as a stress protector. One, as reported for other siderophores, is the prevention of Fenton reaction simply by chelating iron and therefore turning it unavailable [15, 24, 32, 33]. We found that enterobactin protection against oxidative stress occurs in the cell cytoplasm. To reach the cytoplasm, enterobactin has to chelate iron and subsequently be internalized by a specific transport machinery [1]. Once in the cytoplasm, enterobactin is already chelating an iron atom and therefore it is unavailable for further chelation. Moreover, we observed that protection against oxidative stress by enterobactin requires the Fes-mediated cytoplasmic hydrolysis that cleaves the enterobactin backbone, turns iron susceptible to reduction and thus adds one iron atom to the cytoplasmic iron pool [34]. Given that enterobactin is actually providing iron instead of depriving the cell from it, we believe the first scenario is unfeasible. Considering this, there must be a chemical trait of enterobactin that justifies our experimental observations. Catecholate molecules have low radical potentials and therefore act as hydrogen atom donors allowing the termination of radical chain reactions [35]. Then, the second scenario would be that after iron release, the cleaved enterobactin backbone exposes three catecholate moieties with the freed hydroxyl groups available for ROS scavenging. To test if structural features of enterobactin could be responsible for the antioxidant activity, we used the *in vitro* 2,2-diphenyl-1-picrylhydrazyl (DPPH) scavenging system. The results presented in Fig 6A show that the iron-free form of enterobactin exhibited high scavenging activity and that this effect was dose-dependent. In this experimental setting, 10 μM of enterobactin was able to scavenge a 100% of DPPH in a concentration of 100 μM. As controls we used a well-known antioxidant, ascorbic acid, and the catechol-carrying molecule 2,3 dihydroxybenzoic acid (DHBA). For all concentrations assayed, enterobactin showed higher antioxidant activity than DHBA and ascorbic acid (Fig 6A). The lower scavenging activity of DHBA not only validates the ability of catechol moieties to reduce DPPH but also evidences that the number of them in each molecule determine the overall antioxidant capacity. Furthermore, the addition of Fe^+3^ to the reaction significantly decreased the enterobactin scavenging activity (Fig 6B). Thus, we hypothesize that this reduced scavenging activity is a consequence of the iron binding which occupies the hydroxyl groups. This is consistent with our *in vivo* observations in which enterobactin has to be hydrolyzed by Fes in order to protect from oxidative stress [19].

There is increasing evidence that siderophores may play alternative roles, aside of providing cells with the necessary iron [36]. These roles most likely rely on the chemical diversity found in siderophores. Even though the core chemical structure potentially involves catechol, hydroxamate, phenolate, thiazolidine or carboxylate groups, more than 500 chemically different siderophores have been reported so far [37]. Interestingly, some uropathogenic *E*. *coli* strains produce the siderophores enterobactin (catechol), salmochelin (catechol), aerobactin (hydroxamate) and yersiniabactin (thiazolidine) [38]. In addition, these bacteria can use heterologous siderophores produced by other microorganisms [2]. The accumulation of large gene clusters for the synthesis and genes for uptake of several siderophores is an apparent redundancy from an energy conservation perspective. However, that would not be the case if siderophores have alternative roles that make cells more resourceful to adapt to a changing environment.

### Concluding remarks

In this work, we demonstrate that enterobactin protects intracellularly against oxidative stress and that this protection is independent of iron availability. Accordingly, we observed that enterobactin transcriptional expression and production is under a dual control: the classical iron repression and a novel mechanism involving oxidative stress induction. The ROS protection effect would be a result of the ability of enterobactin to directly stabilize radicals through its hydroxyl moieties bound to the aromatic rings. Then, this iron uptake system would ensure *E*. *coli* not only iron entry into the cell, which might lead to oxidative stress through Fenton reaction, but the simultaneous generation of a molecule that scavenges ROS and therefore counterbalances the potential damage that iron excess might cause. This role of enterobactin could help the microorganism to survive in nature and therefore have strong implications in microbial ecology. Altogether, recent discoveries in siderophore alternative functions portray a scenario where iron assimilation might be only “the tip of the iceberg”.

## Materials and Methods

### Bacterial strains and Growth conditions

The strains and plasmids employed in this work are listed in Table 2. Strains were grown in aerobic conditions at 37°C in M9 (Sigma-Aldrich) medium supplemented with 0.2% casamino acids, 0.2% glucose, 1 mM MgSO_4_ and 1 mg/mL vitamin B1. Solid media contained 1.5% agar. Antibiotics (Kanamycin 50 μg/mL, Ampicilin 50 μg/mL) were added when required.

**Table 2:** List of strains and plasmids used in this work.

**Strains**	**Relevant genotype**	**Source**
*Escherichia coli* BW25113	wild type	CGSC^a^
*Escherichia coli* JW0586-1	BW25113 Δ*entE*::kan	CGSC^a^
*Escherichia coli* CA10	BW25113 Δ*entE*::*lacZ*	This work
*Escherichia coli* JW0588-1	BW25113 Δ*entA*::kan	CGSC^a^
*Escherichia coli* JW0587-1	BW25113 Δ*entB*::kan	CGSC^a^
*Escherichia coli* JW0578-1	BW25113 Δ*entF*::kan	CGSC^a^
*Escherichia coli* JW0581-3	BW25113 Δ*fepG*::*kan*	CGSC^a^
*Escherichia coli* JW0576-2	BW25113 Δ*fes*::*kan*	CGSC^a^
*Escherichia coli* JW0584-1	BW25113 Δ*fepB*::*kan*	CGSC^a^
*Escherichia coli* JW0580-5	BW25113 Δ*fepC*::*kan*	CGSC^a^
*Escherichia coli* JW0582-2	BW25113 Δ*fepD*::*kan*	CGSC^a^
*Escherichia coli* JW4023-5	BW25113 Δ*soxS*::*kan*	CGSC^a^
*Escherichia coli* DRP1	BW25113 Δ*entE*::*lacZ* Δ*soxS*	This work
**Plasmids**		
pNTR-*entE*	pNTR-SD carrying *entE*	Saka *et al*. (2005) [39]
pALM23	*ryhB-lacZ* transcriptional fusion in pQ50	Ma & Payne (2002) [25]

^**a**^**CGSC,** *Escherichia coli* Genetic Stock Center.

### General methods

Plasmid DNA was isolated using the Wizard Miniprep DNA purification system (Promega) according to the manufacturer’s instructions. Transformation of competent cells was performed as described previously [40] and transductions involved the use of the bacteriophage P1vir following the method described by Miller (1992) [41].

### Construction of BW25113 Δ*entE*::*lacZ* and Δ*entE*::*lacZ* Δ*soxS* strains

The resistance cassettes of BW25113 Δ*entE*::kan and Δ*soxS*::kan were removed using the FLP recombinase encoded in thermosensitive plasmid pCP20 [42]. The process generated unmarked Δ*entE* and Δ*soxS* deletions. The chromosomal transcriptional fusion Δ*entE*::*lacZ* was generated on a Δ*entE* strain using the pKG136 plasmid, according to the procedure developed by Ellermeier *et al*. (2002) [43]. To obtain a Δ*entE*::*lacZ* Δ*soxS* strain, Δ*entE*::*lacZ* fusion was transduced into a Δ*soxS* mutant.

### Sensitivity assays

To perform the sensitivity assay, 50 μl of overnight cultures were mixed with 5 mL of soft agar (0.8% agar) and overlaid onto M9 agar medium (M9A) plates supplemented with 100 μM FeCl_3_ (Sigma-Aldrich), 5mM ascorbic acid (Sigma-Aldrich) or 1μM enterobactin (EMC micro collections). Then a sterile filter paper disc containing 5 μL of 9.98 M H_2_O_2_ (Cicarelli Laboratories) or 5 μL of 1 M paraquat (Sigma-Aldrich.) was placed in the center of the plate. Zones of growth inhibition were measured after overnight incubation at 37°C.

### β-Galactosidase assays

The β-galactosidase activity was determined following the method described by Zhou & Gottesman (1998) [44] with modifications. Strains carrying the *entE*::*lacZ* or *ryhB*::*lacZ* transcriptional fusions were grown in M9 medium at 37°C with aeration up to OD_600nm_ = 0.8. At this point the culture was divided in equal parts, one aliquot was used as control and the others were incubated with 100 μM paraquat (PQ), 1mM hydrogen peroxide (H_2_O_2_), 5 mM ascorbic acid (ASC), 100 μM FeCl_3_ or a combination of these compounds. Samples were incubated at 37°C with aeration and after two hours, β-galactosidase activity was determined. For this, 600 μL of these cultures were permeabilized with 0.1% SDS (24 μL) and chloroform (48 μL) for 20 min. Then, 100 μL of permeabilized cells were placed on 96-well microtiter plate and 100 μL of a 1.32 mg/mL solution of 2-nitrophenyl- β -D-galactopyranoside in buffer Z was added. Finally, the absorbance at 420 nm was measured for 20 min in a SpectraMax 250 spectrophotometer. Specific activity was calculated by dividing the slope of the line over time by the corresponding OD_600nm_ and expressed as arbitrary units (AU).

### Enterobactin-enriched extracts

Enterobactin was partially purified from a BW25113 wild-type culture supernatant following the protocol described by Winkelmann *et al*. 1994 [45]. Briefly, cells were grown for 20 hours at 37°C in M9 medium supplemented with 0.2% casamino acids, 0.2% glucose, 1 mM MgSO_4_ and 1 mg/mL vitamin B1 with the addition of 1mM H_2_O_2_, 25 μM FeCl_3_ or 1mM H_2_O_2_ and 25 μM FeCl_3_ simultaneously. The cell free supernatant was acidified to pH 2 using 5 N HCl and enterobactin was extracted once adding an equal volume of ethyl acetate and mixing with a magnetic stirrer for 30 min. The organic fraction was collected, dried *in vacuo* and resuspended in methanol.

### Determination of catechol siderophore production

Catechol production was estimated using the colorimetric Arnow assay and a biological assay based on the reversion of a colony development arrest phenotype.

Arnow assay was carried out according to the method described by Arnow *et al*. (1937) [46]. Briefly, 1 mL 0.5 N HCl, 1 mL nitrite-molybdate (1g sodium nitrite and 1 g sodium molybdate dissolved in 10 mL water), 1 mL 1 N NaOH and 1 mL H_2_O were added to 1 mL of the enterobactin-enriched extract. Finally, the absorbance at 510 nm was determined in order to quantify catechol siderophore concentration. Results were expressed as micromolar equivalents by comparison to a standard curve prepared using 2,3 dihydroxybenzoic acid (DHBA) (Sigma).

The colony arrest reversion assay was designed based on the observations made by Adler *et al*. (2014) [19]. For that, 100 μL of a 10^−5^ dilution from an *entE* overnight culture were spread on the surface of agarized M9 medium and incubated overnight at 37°C. No colony development was obtained after this incubation. Then, 5 μL of double serial dilutions of extracts having enterobactin were spotted on the surface and plates were reincubated and screened for colony formation. The higher dilution that allowed colony formation was documented. Assays were made at least in triplicates.

### Evaluation of antioxidant property: DPPH radical scavenging activity

*In vitro* radical scavenging activity of enterobactin was determined using 2,2-diphenyl-1-picrylhydrazyl (DPPH) radical (Sigma-Aldrich) following the method described by Brand-Williams *et al*. (1995) [47]. An aliquot of 900 μL of pure enterobactin or ascorbic acid at different concentrations (1–50 μM) were mixed with 100 μL of 1 mM DPPH in methanol. Methanol was used as control for zero scavenging activity. Solutions were incubated in dark at room temperature for 30 min. As an indication of DPPH reduction, absorbance at 517 nm was measured. The percentage of reduced DPPH was calculated using the following equation:

$$DPPH\;scavenging\;activity\;(\%)=\frac{Abs\;control–Abs\;sample}{Abs\;control}\times 100$$

To estimate the ability of enterobactin to scavenge radicals when chelating iron, 5 μM enterobactin aliquots with or without 5 μM FeCl_3_ were mixed with 100 μL of 1 mM DPPH. Absorbance was measured at 517 nm after 30 min of incubation at room temperature. FeCl_3_ was used as a negative control to confirm that iron did not react with DPPH.

## Supporting Information

S1 Fig Enterobactin gene cluster in *Escherichia coli*. Genes are tightly organized into six operons originating from three Fur-controlled bidirectional promoter-operator regions. These regions are located between *fepB* and the *entCEBAH* operon, between *fepD* and *entS* and finally between *fepA* and *fes*. Promoters are depicted with arrows and Fur boxes are represented by red squares.
(TIF)

S2 Fig Representative images depicting sensitivity assays. Figure shows the zone of clearance obtained with H_2_O_2_ (Panel A) and paraquat (Panel B) for wild-type *E*. *coli* and the *entE* mutant. pNTR-*entE*, ENT, ASC, FeCl_3_ indicates complementation with a plasmid harboring the *entE* gene, medium supplementation with 1 μM of pure enterobactin, 5 mM ascorbic acid and 100 μM FeCl_3_ respectively.
(TIF)

S3 Fig H_2_O_2_ and paraquat sensitivity of additional *E*. *coli* mutants impaired in enterobactin synthesis. Data is plotted as the mean values ± standard deviation (SD) of the zone of clearance obtained with strains *entA* (Panel A), *entB* (Panel B) and *entF* (Panel C) relative to the zone of clearance of the wild-type strain in M9 medium. ENT, ASC and FeCl_3_ indicate medium supplementation with 1 μM enterobactin, 5 mM ascorbic acid and 100 μM FeCl_3_, respectively. Experiments were done in triplicates.
(TIF)

S4 Fig H_2_O_2_ and paraquat sensitivity of additional *E*. *coli* mutants impaired in enterobactin uptake. Data is plotted as the mean values ± standard deviation (SD) of the zone of clearance obtained with strains *fepB* (Panel A), *fepC* (Panel B) and *fepD* (Panel C) relative to the zone of clearance of the wild-type strain in M9 medium. ENT, ASC and FeCl_3_ indicate medium supplementation with 1 μM enterobactin, 5 mM ascorbic acid and 100 μM FeCl_3_, respectively. Experiments were done in triplicates.
(TIF)
