# Supplementary figures and images for: Enterobactin as Part of the Oxidative Stress Response Repertoire
Source: PLoS One. 2016 Jun 16;11(6):e0157799. doi: 10.1371/journal.pone.0157799 (PMC4911079; doi:10.1371/journal.pone.0157799)

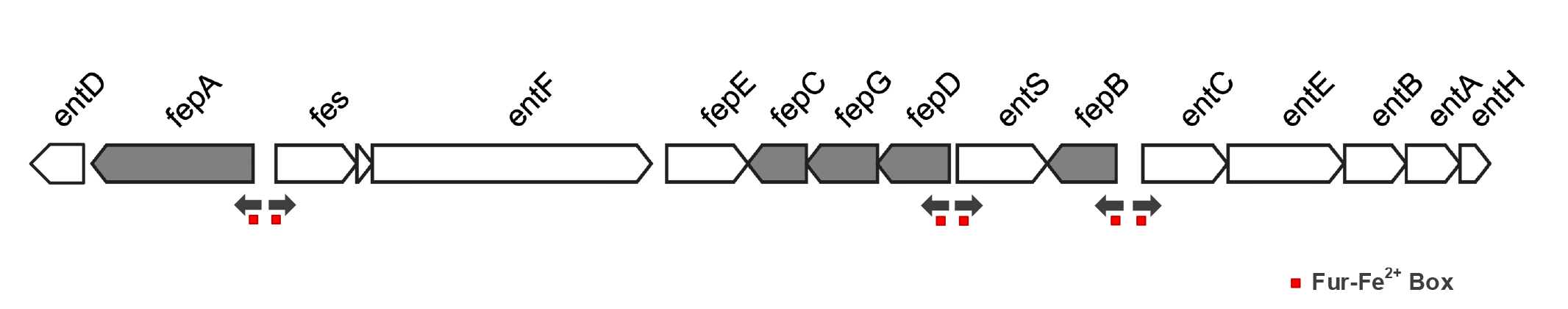

Supplement: S1 Fig — Genes are tightly organized into six operons originating from three Fur-controlled bidirectional promoter-operator regions. These regions are located between fepB and the entCEBAH operon, between fepD and entS and finally between fepA and fes. Promoters are depicted with arrows and Fur boxes are represented by red squares. (TIF) [file pone.0157799.s001.tif]

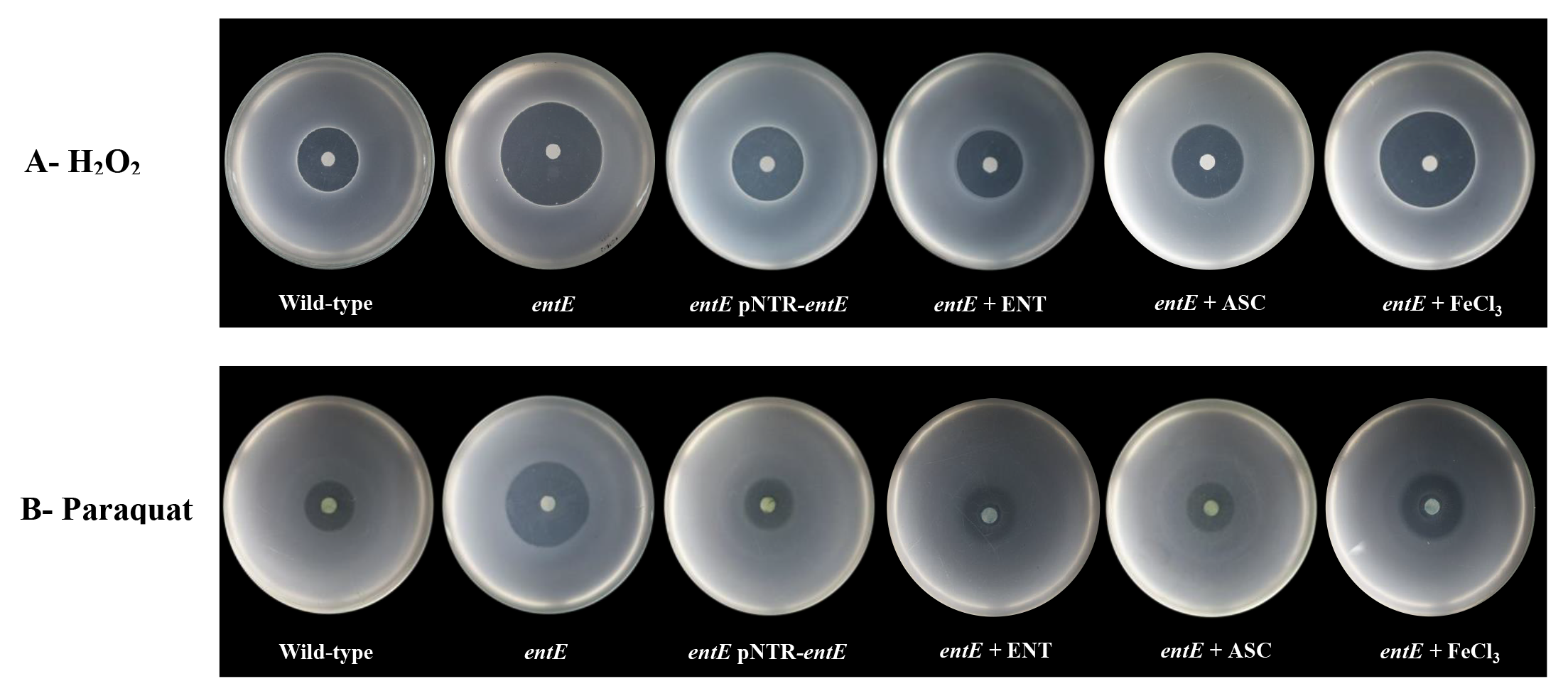

Supplement: S2 Fig — Figure shows the zone of clearance obtained with H2O2 (Panel A) and paraquat (Panel B) for wild-type E. coli and the entE mutant. pNTR-entE, ENT, ASC, FeCl3 indicates complementation with a plasmid harboring the entE gene, medium supplementation with 1 μM of pure enterobactin, 5 mM ascorbic acid and 100 μM FeCl3 respectively. (TIF) [file pone.0157799.s002.tif]

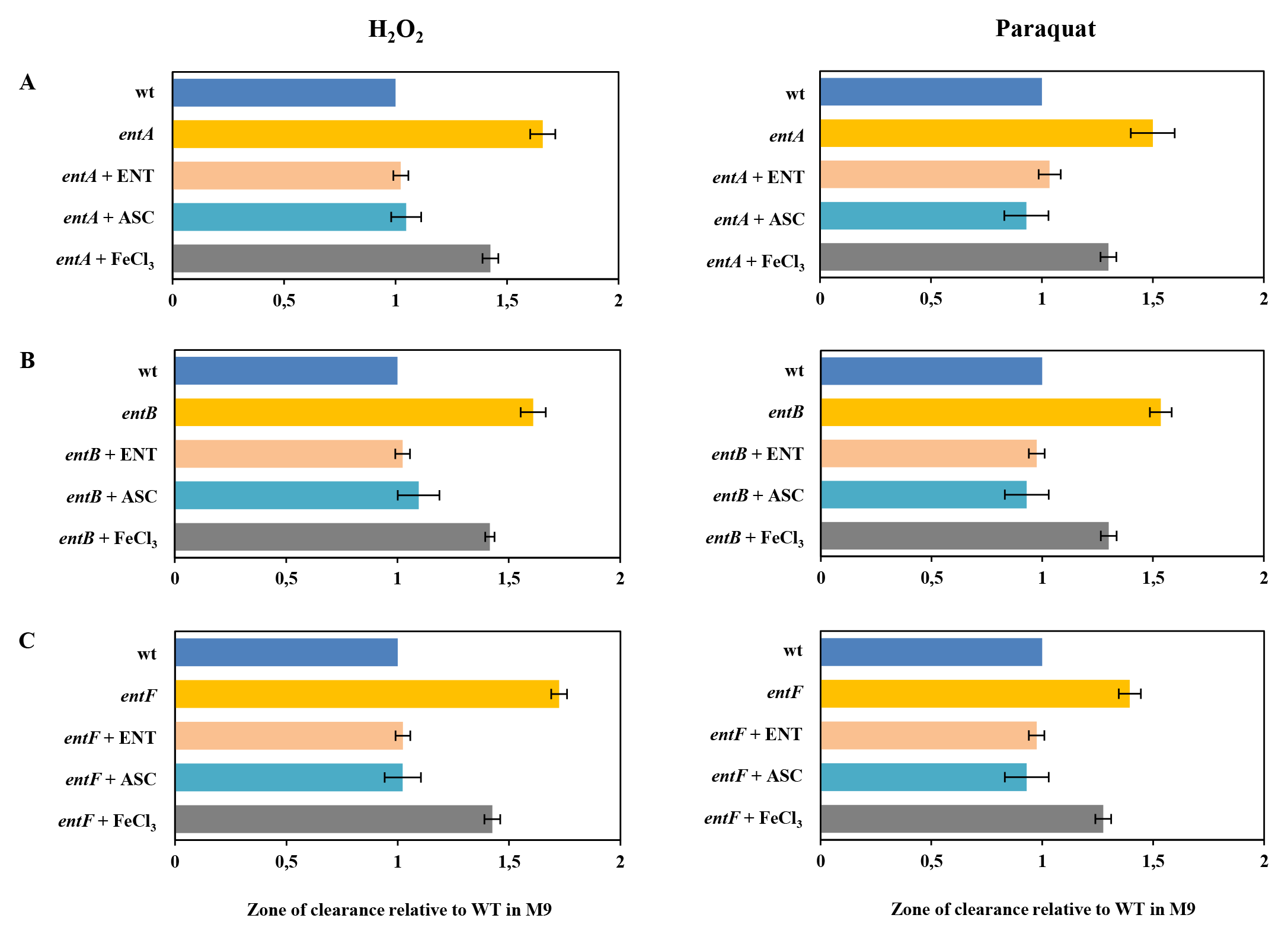

Supplement: S3 Fig — Data is plotted as the mean values ± standard deviation (SD) of the zone of clearance obtained with strains entA (Panel A), entB (Panel B) and entF (Panel C) relative to the zone of clearance of the wild-type strain in M9 medium. ENT, ASC and FeCl3 indicate medium supplementation with 1 μM enterobactin, 5 mM ascorbic acid and 100 μM FeCl3, respectively. Experiments were done in triplicates. (TIF) [file pone.0157799.s003.tif]

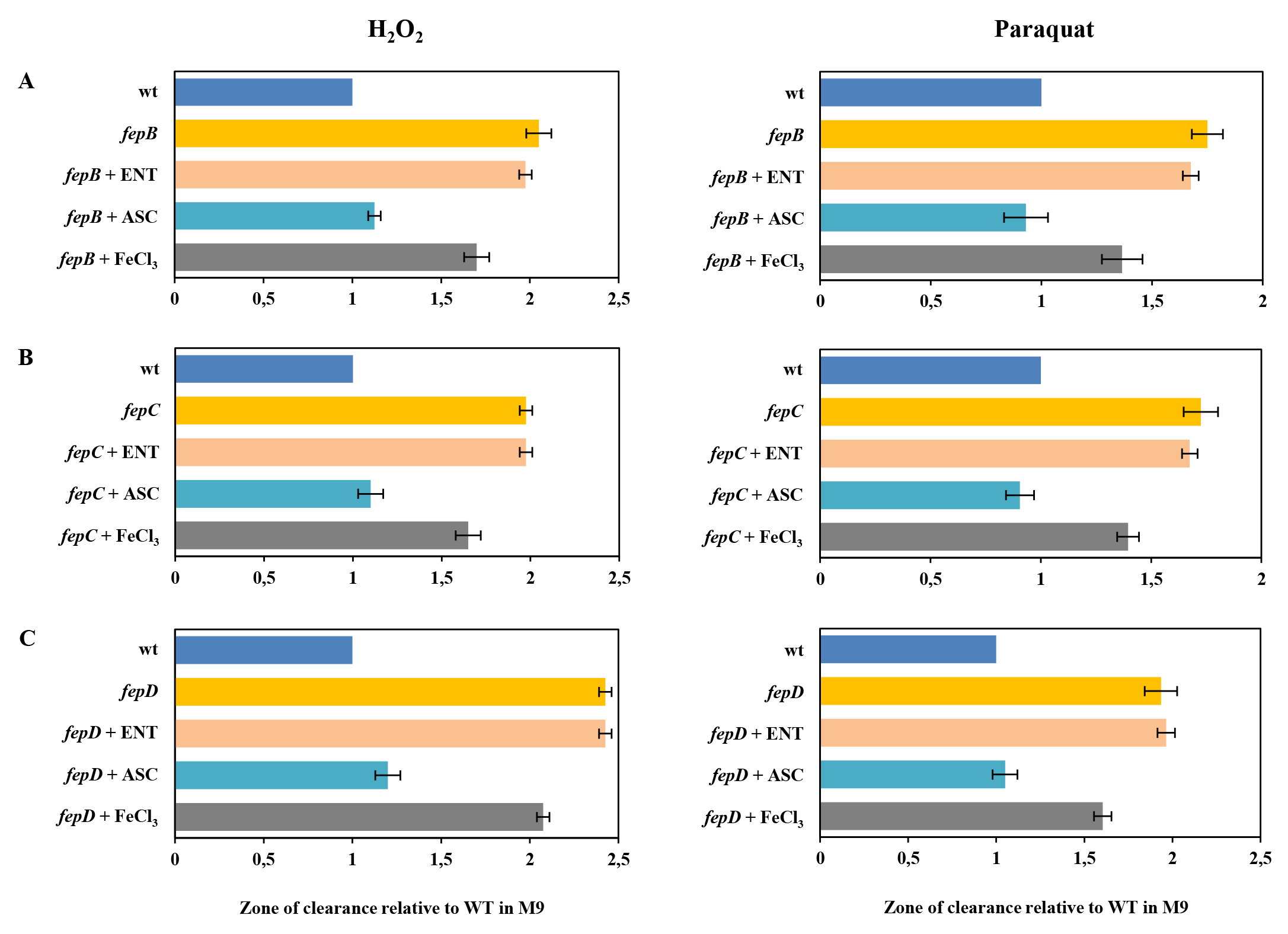

Supplement: S4 Fig — Data is plotted as the mean values ± standard deviation (SD) of the zone of clearance obtained with strains fepB (Panel A), fepC (Panel B) and fepD (Panel C) relative to the zone of clearance of the wild-type strain in M9 medium. ENT, ASC and FeCl3 indicate medium supplementation with 1 μM enterobactin, 5 mM ascorbic acid and 100 μM FeCl3, respectively. Experiments were done in triplicates. (TIF) [file pone.0157799.s004.tif]
